# 2-Methoxyestradiol as an Antiproliferative Agent for Long-Term Estrogen-Deprived Breast Cancer Cells

**DOI:** 10.3390/cimb45090464

**Published:** 2023-09-09

**Authors:** Masayo Hirao-Suzuki, Koki Kanameda, Masufumi Takiguchi, Narumi Sugihara, Shuso Takeda

**Affiliations:** 1 Laboratory of Xenobiotic Metabolism and Environmental Toxicology, Faculty of Pharmaceutical Sciences, Hiroshima International University, 5-1-1 Hiro-koshingai, Kure-shi 737-0112, Hiroshima, Japan; m-hirao@hirokoku-u.ac.jp (M.H.-S.); m-takigu@hirokoku-u.ac.jp (M.T.); 2 Laboratory of Molecular Life Sciences, Faculty of Pharmacy and Pharmaceutical Sciences, Fukuyama University, Sanzou 1, Gakuen-cho, Fukuyama-shi 729-0292, Hiroshima, Japan; p7118025@fukuyama-u.ac.jp (K.K.); sugihara@fukuyama-u.ac.jp (N.S.)

**Keywords:** 2-methoxyestradiol, 2-MeO-E2, LTED cells, tubulin, G-1, breast cancer

## Abstract

To identify effective treatment modalities for breast cancer with acquired resistance, we first compared the responsiveness of estrogen receptor-positive breast cancer MCF-7 cells and long-term estrogen-deprived (LTED) cells (a cell model of endocrine therapy-resistant breast cancer) derived from MCF-7 cells to G-1 and 2-methoxyestradiol (2-MeO-E2), which are microtubule-destabilizing agents and agonists of the G protein-coupled estrogen receptor 1 (GPER1). The expression of GPER1 in LTED cells was low (~0.44-fold), and LTED cells displayed approximately 1.5-fold faster proliferation than MCF-7 cells. Although G-1 induced comparable antiproliferative effects on both MCF-7 and LTED cells (IC_50_ values of >10 µM), 2-MeO-E2 exerted antiproliferative effects selective for LTED cells with an IC_50_ value of 0.93 μM (vs. 6.79 μM for MCF-7 cells) and induced G2/M cell cycle arrest. Moreover, we detected higher amounts of β-tubulin proteins in LTED cells than in MCF-7 cells. Among the *β-tubulin* (*TUBB*) isotype genes, the highest expression of *TUBB2B* (~3.2-fold) was detected in LTED cells compared to that in MCF-7 cells. Additionally, siTUBB2B restores 2-MeO-E2-mediated inhibition of LTED cell proliferation. Other microtubule-targeting agents, i.e., paclitaxel, nocodazole, and colchicine, were not selective for LTED cells. Therefore, 2-MeO-E2 can be an antiproliferative agent to suppress LTED cell proliferation.

## 1. Introduction

**Figure 1:**
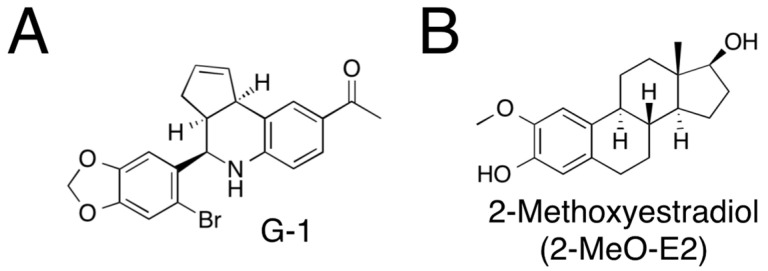
The structures of G-1 (**A**) and 2-methoxyestradiol (2-MeO-E2) (**B**).

Estrogen receptors (ERs) are pivotal in breast cancer development and progression, and their expression and activity are tightly regulated. Among patients with breast cancer, >70% are ER-positive at diagnosis. Clinically, ER-positive patients are treated with agents such as aromatase inhibitors and fulvestrant (also known as ICI 182780, an antagonist of ERα), which exert estrogen-lowering effects by suppressing estrogen signaling [1]. Although >50% of patients are sensitive to the medications, most patients exhibit relapse [1]. Thus, resistance to endocrine therapy remains a major clinical concern in breast cancer, and understanding the molecular mechanism(s) responsible for the acquired resistance to endocrine therapy is important. An experimental cell model (i.e., long-term estrogen-deprived [LTED] cells) was established by maintaining parental human breast cancer MCF-7 cells (ER-positive) under estrogen-deprived conditions for approximately six months [2,3,4]. In addition to the previously reported feature that ERs expressed in LTED cells display ligand-independent activity [2,3,4,5,6], we recently reported that LTED cells express very low levels of G protein-coupled estrogen receptor 1 (GPER1, formerly known as GPR30), such that its expression is comparable to that of GPER1 produced by human breast cancer MDA-MB-231 cells, a GPER1-negative (or very low), and an ER-negative cell line [6]. GPER1 is a membrane-type receptor discovered in 1996 in breast cancer tissue [7]. G-1 (also known as LNS-8801) (Figure 1A), a GPER1-selective agonist, was identified in 2006 by Bologa et al. and has been shown to exert no significant effects on other protein-coupled receptors or ERα/β [8]. The biological impact of G-1 treatment on breast cancer cell proliferation and survival is controversial, although G-1-mediated stimulation of breast cancer cells has been reported [9,10,11,12,13]. However, we and others demonstrated that G-1 abrogates the proliferation of both MCF-7 (ER/GPER1-positive) and MDA-MB-231 (ER/GPER1-negative) breast cancer cells, with more substantial effects on MDA-MB-231 cells than on MCF-7 cells [6,14].

Wang’s research group has reported that G-1 can also act as a microtubule-destabilizing agent and target β-tubulins to kill MDA-MB-231 and MCF-7 cells [14,15]. Similar to G-1, 2-methoxyestradiol (2-MeO-E2) (Figure 1B), a metabolite of 17β-estradiol, has been shown to bind to the colchicine-binding site on β-tubulins [14,16] and has the potential to activate GPER1 as an agonist [17]. Although E2 metabolites are known to either stimulate or abrogate breast cancer cell proliferation, 2-MeO-E2 can act as an antiproliferative agent in both ER-positive and -negative breast cancer cells [18,19,20]. Microtubules comprise two types of tubulin proteins, α- and β-tubulin, that heterodimerize [21]. α/β-Tubulins comprise multiple isotypes, each encoded by a different gene. Microtubules are targeted for anti-cancer therapies, and a large majority of the United States Food and Drug Administration-approved tubulin inhibitory drugs target β-tubulins. The fluctuating expression of β-tubulin isotypes is profoundly related to resistance to microtubule-targeting agents [22,23]. We and others have reported that compared with parental MCF-7 cells, LTED cells exhibit stronger resistance to several antiproliferative agents, including etoposide (a topoisomerase IIα inhibitor), LY2835219 (a cyclin-dependent kinase 4/6 inhibitor), and trichostatin A (a histone deacetylase inhibitor) [24]. Based on the above-mentioned findings, we analyzed the expression level of β-tubulin and its isotype involved in the aggressive behavior of LTED cells. We sought to investigate whether G-1 and 2-MeO-E2 are effective antiproliferative agents for LTED cells.

## 2. Materials and Methods

### 2.1. Reagents

G-1 (Chemical Abstract Service [CAS] number 881639-98-1, purity ≥ 98%) and 2-MeO-E2 (CAS number 362-07-2, purity ≥ 95%) were purchased from Cayman Chemicals (Ann Arbor, MI, USA). Paclitaxel (CAS number 33069-62-4, purity ≥ 98%) was obtained from FUJIFILM Wako Pure Chemical Corporation (Osaka, Japan). Nocodazole (CAS number 31430-18-9, purity ≥ 99%) was supplied by Sigma-Aldrich (St. Louis, MO, USA), and colchicine (CAS number 64-86-8, purity ≥ 97%) was purchased from Tokyo Chemical Industry Co., Ltd. (Tokyo, Japan). All chemicals were dissolved in dimethyl sulfoxide (molecular biology grade).

### 2.2. Cell Culture, Chemical Treatments, and Cell Morphology Analysis

The cell culture methods were based on previously described procedures [6,25,26]. Briefly, human breast cancer MCF-7 cells (obtained from the American Type Culture Collection, Rockville, MD, USA) were routinely grown in phenol red-containing minimum essential medium α (MEMα) (FUJIFILM Wako Pure Chemical Corporation) supplemented with 10 mM 4-(2-hydroxyethyl)-1-piperazineethanesulfonic acid (HEPES), 5% fetal bovine serum (FBS), 100 U/mL penicillin, and 100 μg/mL streptomycin in a humidified incubator with an atmosphere of 5% CO_2_ at 37 °C. LTED cells were derived from parental MCF-7 cells as previously described [2,3,4,6]. The LTED cell batches obtained were routinely cultured in phenol red-free MEMα (FUJIFILM Wako Pure Chemical Corporation) supplemented with 10 mM HEPES, 5% dextran-coated charcoal-treated FBS (DCC-FBS), 100 U/mL penicillin, and 100 μg/mL streptomycin. Prior to the 24 h chemical treatments, the culture medium was changed to phenol red-free MEMα supplemented with 10 mM HEPES, 5% DCC-FBS, 100 U/mL penicillin, and 100 μg/mL streptomycin. The cells were treated with G-1, paclitaxel, nocodazole, 2-MeO-E2, or colchicine in the culture medium at different concentrations and time points, as indicated in each Figure legend. Cell morphology analysis was performed as previously described [6].

### 2.3. Preparation of Total RNA and Real-Time Reverse Transcription Polymerase Chain Reaction (RT-PCR)

Total RNA was extracted, and RT-PCR was performed as previously described [25,26]. The total RNA concentration was determined using a NanoDrop 2000 UV spectrophotometer (Thermo Fisher Scientific, Waltham, MA, USA). The following PCR primers were used: GPER1 (sense), 5′-TTT GTG GGC AAC ATC CTG ATC-3′; GPER1 (antisense), 5′-CAC CGC CAG GTT GAT GAA GTA-3′; histone deacetylase 6 (HDAC6) (sense), 5′-TGC CTC TGG GAT GAC AGC TT-3′; HDAC6 (antisense), 5′-CCT GGA TCA GTT GCT CCT TGA-3′; the β-tubulin (TUBB) isotypes, TUBB (sense), 5′-CTG CCA CAT CAG TGT TTG AGT C -3′; TUBB (antisense), 5′-AAA AAG ATG GAG GAG GGT TCC-3′; TUBB2A (sense), 5′-GAC GAA CAA GGG GAG TTC G-3′; TUBB2A (antisense), 5′-GGA TGC ACG ATT GAT CTG AG-3′; TUBB2B (sense), 5′-AGG ACG GAC AGA CCC AGA C-3′; TUBB2B (antisense), 5′-CTG ATG ACC TCC CAA AAC TTG-3′; TUBB3 (sense), 5′-GGC CTT TGG ACA TCT CTT CA-3′; TUBB3 (antisense), 5′-CGG TCG GGA TAC TCC TCA-3′; TUBB4A (sense), 5′-TGG TAC ACG GGC GAG GGC AT-3′; TUBB4A (antisense), 5′-GTG GGA AGC GAT GGG AGC AGC-3′; TUBB4B (sense), 5′-CTG CTG CTG TTT GTC TAC TTC C-3′; TUBB4B (antisense), 5′-GCT GAT CAC CTC CCA AAA CT-3′; TUBB6 (sense), 5′-CCA GTT CCT AGC GCA GAG CCG-3′; TUBB6 (antisense), 5′-GCA CGC TGT CCA TGG TGC CT-3′; TUUBB1 (sense), 5′-GGA GAT GAT TGG TGA GGA ACA C-3′; TUBB1 (antisense), 5′-GGT TCT AGG TCC ACC AAG ACT G-3′; β-actin (sense), 5′-GGC CAC GGC TGC TTC-3′; β-actin (antisense), 5′-GTT GGC GTA CAG GTC TTT GC-3′. The primers for human *GPER1*, *HDAC6*, *TUBB*, *TUBB2A*, *TUBB2B*, *TUBB3*, *TUBB4A*, *TUBB4B*, *TUBB6*, *TUBB1,* and *β-actin* were determined in previous reports [6,27,28,29,30,31]. The mRNA levels of *GPER1*, *HDAC6*, *TUBB*, *TUBB2A*, *TUBB2B*, *TUBB3*, *TUBB4A*, *TUBB4B*, *TUBB6*, and *TUBB1* were normalized to that of *β-actin*.

### 2.4. Ponceau Staining and Western Blot Analysis

Antibodies specific to GPER1 (ab39742; Abcam, Cambridge, MA, USA), β-actin (sc-47778; Santa Cruz Biotechnology, Dallas, TX, USA), α-tubulin (017-25031; FUJIFILM Wako Pure Chemical Corporation), and β-tubulin (014-25044; FUJIFILM Wako Pure Chemical Corporation) were used. Whole-cell extracts were prepared, and Western blot analysis was performed as previously described [32]. The protein concentrations of whole-cell extracts were determined using the RC DC^TM^ Protein Assay (Bio-Rad, Hercules, CA, USA). The polyvinylidene difluoride membrane was stained with Ponceau S solution (Beacle Inc., Kyoto, Japan). Pink-stained blots were scanned, and the images were converted to grayscale for publication. Band intensities were quantified using the ImageJ 1.46r software “https://imagej.nih.gov/ij/ (accessed on 21 September 2022)”. The values obtained for the GPER1 band were normalized to the intensity of the β-actin band (internal control).

### 2.5. Cell Proliferation Analysis (MTS Assay) and Cell Counting

MTS assays were performed to determine cell proliferation as previously described [25]. The cells were seeded in 96-well plates at a density of 5 × 10^3^ cells/well. After chemical treatment, cell proliferation was analyzed using the CellTiter 96^®^ AQ_ueous_ One Solution Cell Proliferation Assay Kit (MTS Reagent; Promega, Madison, WI, USA). Cells were counted using a TC10^TM^ Automated Cell counter (Bio-Rad).

### 2.6. Cell Cycle Analysis Using Flow Cytometry

Cells were stained with propidium iodide as previously described [6]. Stained cells were analyzed using a FACSCalibur^TM^ flow cytometer (BD Biosciences, Franklin Lakes, NJ, USA). The obtained data were analyzed using ModFit LT^TM^ v3.0 (Verity Software House, Topsham, ME, USA).

### 2.7. Gene Silencing

To avoid off-target effects, we used commercially available TUBB2B siRNA (sc-105006; Santa Cruz Biotechnology) and a pool of three target-specific siRNAs. Control siRNA (sc-37007; Santa Cruz Biotechnology) was used as the negative control. TUBB2B and control siRNAs were transfected using Lipofectamine RNAiMAX reagent (Thermo Fisher Scientific). The siRNA concentration used for transfection was 15 nM.

### 2.8. Data Analysis

The IC_50_ values were calculated by fitting the dose–response curves using the SigmaPlot 11 software (Systat Software, Inc., San Jose, CA, USA). Differences were considered statistically significant when *p* values were less than 0.05. The statistical significance of the difference between the two groups was determined using Student’s *t*-test. The statistical significance of the differences between multiple groups was determined using analysis of variance (ANOVA) with Dunnett’s or Tukey–Kramer’s post hoc test. These calculations were performed using the StatView 5.0 J software (SAS Institute Inc., Cary, NC, USA).

## 3. Results

### 3.1. On LTED Cells, 2-MeO-E2 Exerts Selective Antiproliferation

**Figure 2:**
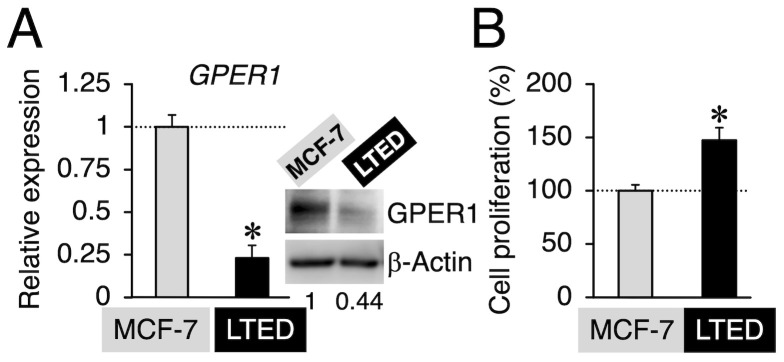
Highly proliferative long-term estrogen-deprived (LTED) cells display a lower expression of G protein-coupled estrogen receptor 1 (GPER1). (**A**) Basal *GPER1* mRNA and GPER1 protein (*inset*) expression in MCF-7 and LTED cells. Data are presented as the mean ± S.E. (*n* = 6) of the fold induction from MCF-7 cells. Significant differences (by Student’s *t*-test) to MCF-7 cells are marked with an asterisk (* *p* < 0.05). (**A**, *inset*) Western blot analysis was performed using antibodies specific for GPER1 and β-actin, respectively. Representative images are shown. The band intensity of GPER1 (MCF-7 lane as 1) was quantified using the ImageJ 1.46r software and normalized to the band intensity of β-actin. Data are shown below the blot image. (**B**) Basal proliferation in MCF-7 and LTED cells. Data are presented as the mean ± S.E. (*n* = 6) percentage of MCF-7 cells. Significant differences (by Student’s *t*-test) to MCF-7 cells are marked with an asterisk (* *p* < 0.05).

**Figure 3:**
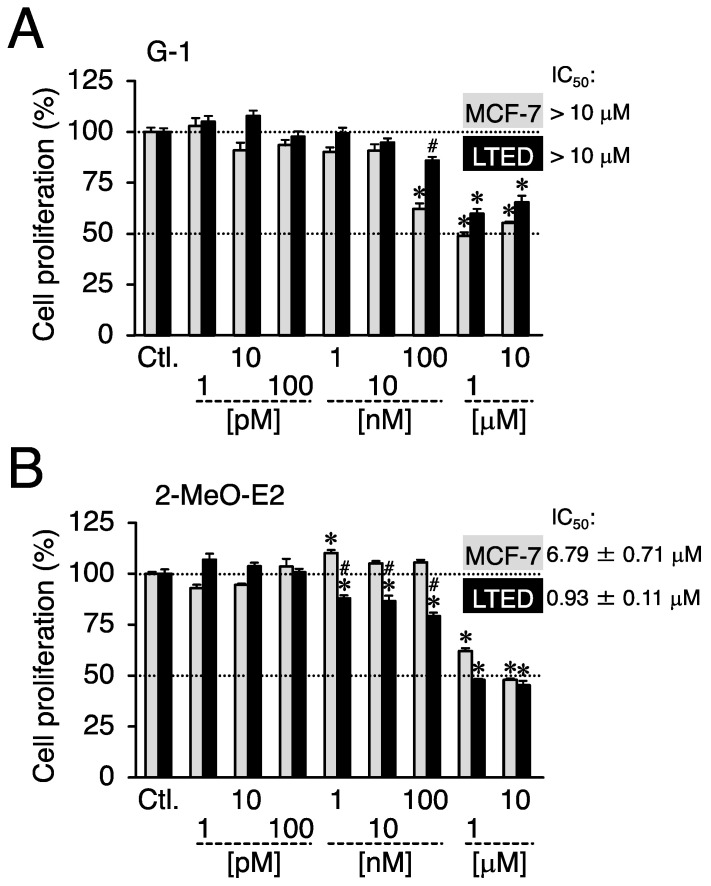
On LTED cells, 2-methoxyestradiol (2-MeO-E2) exerted selective antiproliferative effects. MCF-7 and LTED cells were treated with G-1 (1 pM to 10 μM) (**A**) or 2-MeO-E2 (1 pM to 10 μM) (**B**) for 48 h. The control sample (indicated as Ctl.) cells were treated with the vehicle. Data are presented as the mean ± S.E. (*n* = 6) percentage of the vehicle-treated control. Significant differences (by two-way ANOVA, followed by Tukey–Kramer’s post hoc test) as compared with the vehicle-treated control for each cell and compared with MCF-7 cells are marked with asterisks (* *p* < 0.05) and hashes (^#^ *p* < 0.05), respectively.

**Figure 4:**
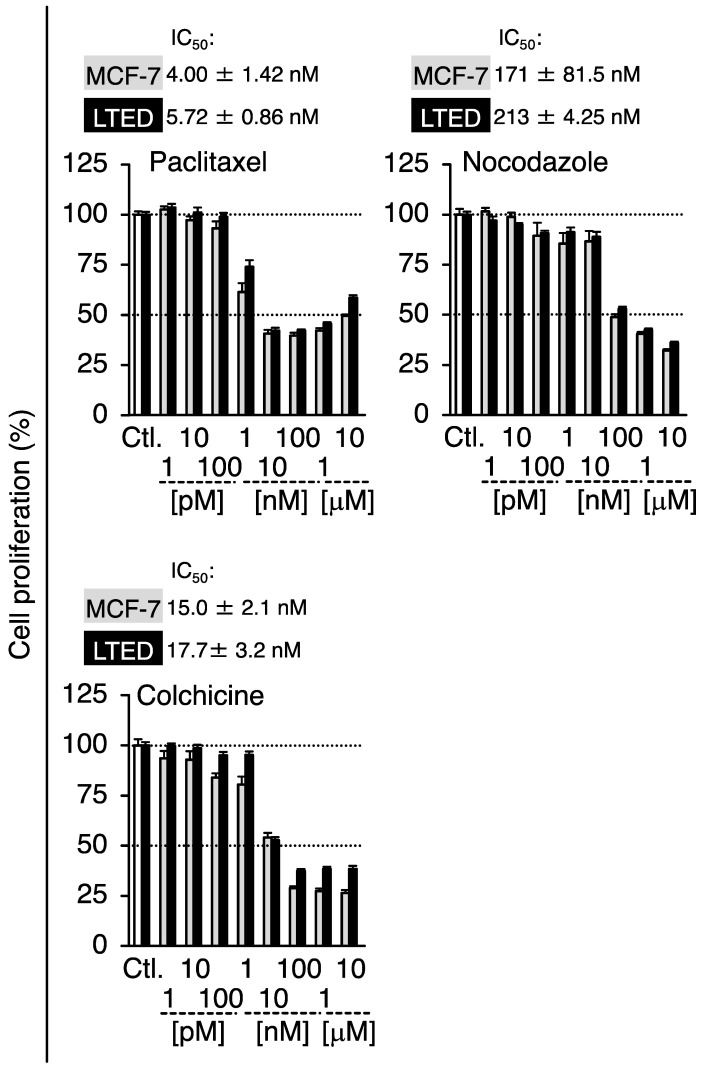
Effects of paclitaxel, nocodazole, and colchicine on the proliferation of MCF-7 and LTED cells. MCF-7 and LTED cells were treated with paclitaxel (**upper left panel**), nocodazole (**upper right panel**), or colchicine (**lower left panel**) (1 pM to 10 μM) for 48 h. The control sample (Ctl.) was treated with the vehicle. Data are presented as the mean ± S.E. (*n* = 6) percentage of the vehicle-treated control.

Prior to conducting experiments to determine whether G-1 and 2-MeO-E2 negatively affected the proliferation of LTED and MCF-7 cells by targeting GPER1, we confirmed the expression profile of GPER1 in these breast cancer cells. The mRNA expression level of *GPER1* was less than that (0.23-fold, *p* < 0.05) in LTED cells compared to that in parental MCF-7 cells (Figure 2A). Consistent with the mRNA expression profile, GPER1 protein expression displayed a similar trend (0.44-fold) (Figure 2A, *inset*) [6]. As shown in Figure 2B, LTED cells exhibited more aggressive proliferative features than MCF-7 cells. The reduced expression of GPER1 favors cell aggressiveness, such as stimulated cell proliferation, particularly in LTED cells, as indicated in the reports suggesting that GPER1 can act as a tumor suppressor in different types of cancers, including breast cancer [33,34,35,36]. Therefore, the possible negative (antiproliferative) effects of GPER1 agonists (G-1 and 2-MeO-E2) [8,17] on the aggressive behavior of LTED cells were not observed or weakened. Therefore, we investigated the validity of this hypothesis. Both LTED and MCF-7 cells were exposed to G-1 and 2-MeO-E2 in varying concentrations from 1 pM to 10 μM for 48 h; 2-MeO-E2 exhibited potent/selective inhibitory effects on the proliferation of LTED cells as compared with that on MCF-7 cells (IC_50_ values: 6.79 ± 0.71 and 0.93 ± 0.11 μM, respectively) (Figure 3A,B). Furthermore, the antiproliferative effects of 2-MeO-E2 on LTED cells were prolonged in a time-dependent manner for up to 96 h, with lower IC_50_ values of 0.55 ± 0.02 and 0.40 ± 0.02 μM observed at 72 and 96 h, respectively (Supplemental Figure S1). To confirm the antiproliferative selectivity of 2-MeO-E2 in LTED cells, we investigated other representative microtubule-destabilizing agents (i.e., paclitaxel, nocodazole, and colchicine) belonging to the same group as G-1 and 2-MeO-E2 [24,37]. Although they exhibited stronger antiproliferative effects than 2-MeO-E2, none of the antiproliferative agents displayed selective inhibitory potential for the proliferation of LTED cells (Figure 4). Thus, among the molecules that interact with tubulins, it is strongly suggested that 2-MeO-E2 is a selective antiproliferative molecule for LTED cells.

### 3.2. G-1 Arrests LTED Cells at the G2/M Phase with Accompanying Sub-G1

**Figure 5:**
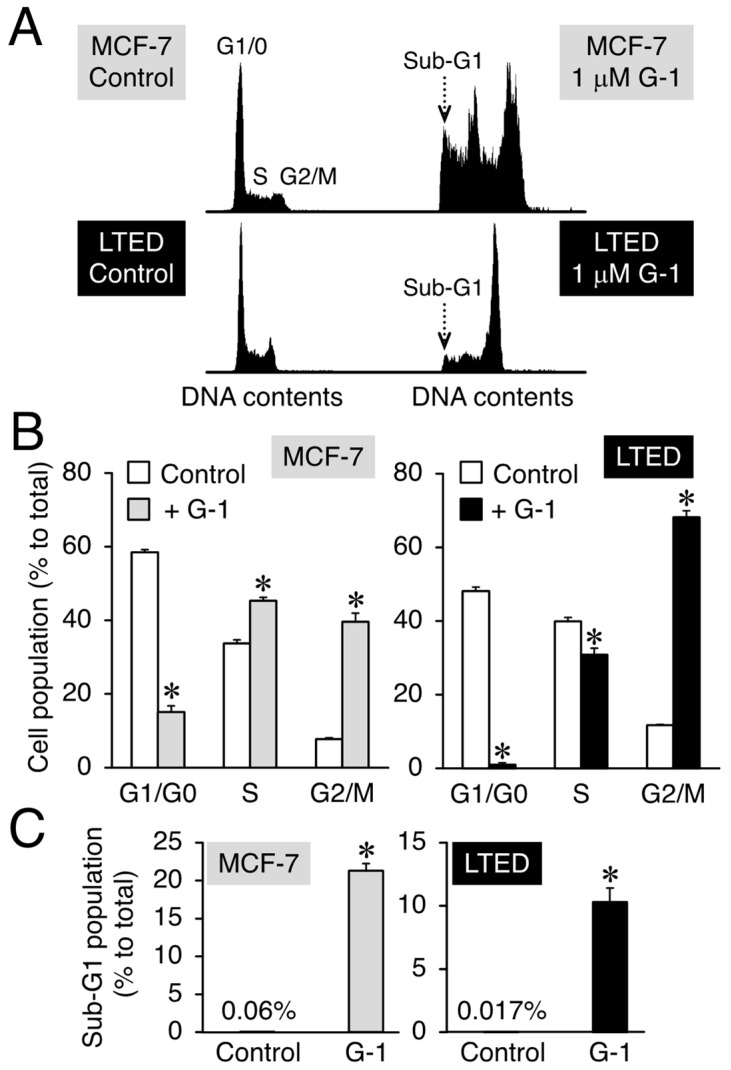
G-1 arrests LTED cells at the G2/M phase with accompanying sub-G1. MCF-7 and LTED cells were treated with 1 μM G-1 for 48 h. The control sample was treated with the vehicle. (**A**) Representative histograms are shown. (**B**,**C**) The percentages of cells in G1, S, G2/M (**B**), and sub-G1 (**C**) phases are shown. Data are presented as the mean ± S.E. (*n* = 3). Significant differences (by Student’s *t*-test) as compared with the vehicle-treated control are marked with asterisks (* *p* < 0.05).

Depending on the concentration used, G-1 can evoke suppressive effects on the proliferation of MCF-7 cells, coupled with apoptosis [6,14]. Furthermore, G-1 decreased the percentage of the cell population in the G1 phase and increased the number of MCF-7 cells in the G2/M phase [14]. Based on these findings, we first performed flow cytometry analysis to study the LTED cell cycle progression after exposure to 1 μM G-1 for 48 h and used MCF-7 cells as a positive control. The flow cytometry results showed that G-1 induced G1 phase downregulation and G2/M phase stimulation in MCF-7 cells compared to that in the vehicle-only treated control (Figure 5A, upper panel; Figure 5B, left panel). We focused on the interplay between G-1 and LTED cells and found that the cell population exhibited characteristics similar to those of MCF-7 cells after exposure to G-1 (Figure 5A, lower panel; Figure 5B, right panel). G-1 induced a population of cells in the sub-G1 phase (a hallmark of apoptosis) in both breast cancer cell lines. The fold induction of the sub-G1 population after G-1 treatment was approximately 355-fold (0.06% vs. 21.3% in MCF-7 cells) and 617-fold (0.017% vs. 10.3% in LTED cells) higher than that in the controls (Figure 5A,C). Cellular morphology analysis after exposure to 1 μM G-1 for 48 h indicated membrane blebbing. One of the apoptotic cell markers [38] was detected in both MCF-7 and LTED cells (Supplementary Figure S2). Considering the significant appearance of the sub-G1 peak after G-1 exposure, G-1 treatment was presumed to decrease the proliferation of LTED cells and induce apoptotic signaling, as previously observed in MCF-7 cells [14].

### 3.3. 2-MeO-E2 Arrests LTED Cells at the G2/M Phase without Accompanying Sub-G1

**Figure 6:**
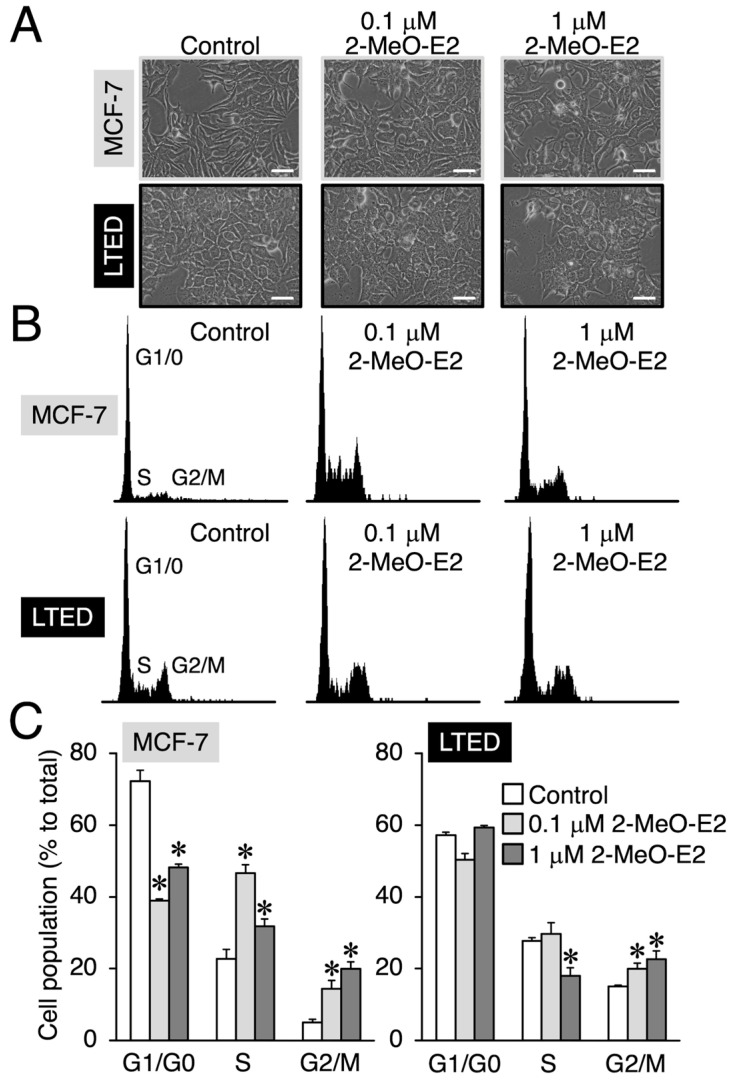
At the G2/M phase, 2-MeO-E2 arrests LTED cells without accompanying sub-G1. MCF-7 and LTED cells were treated with 2-MeO-E2 (0.1 and 1 μM) for 48 h. The control sample was treated with the vehicle. (**A**) Morphology of 2-MeO-E2-treated cells. Representative images are shown. The images were acquired at 400× magnification. Scale bar is indicated as 50 μm. (**B**) Representative histograms are shown. (**C**) The percentage of cells in the G1, S, and G2/M phases are shown. Data are presented as the mean ± S.E. (*n* = 3). Significant differences (by one-way ANOVA, followed by Dunnett’s post hoc test) as compared with the vehicle-treated control are marked with asterisks (* *p* < 0.05).

Cell morphology and cell cycle analyses of MCF-7 and LTED cells exposed to 0.1 μM or 1 μM 2-MeO-E2 revealed differences in the morphology of MCF-7/LTED cells exposed to G-1 and 2-MeO-E2 (Supplementary Figure S2 and Figure 6A). Similar to the effects of G-1, 2-MeO-E2 induced cell cycle arrest at the G2/M phase and reduced the number of MCF-7 cells in the G1/G0 phase (Figure 6B,C) [14,39]. However, unlike the effects of G-1 on MCF-7 and LTED cells (Figure 5 and Supplementary Figure S2), 2-MeO-E2 did not affect the G1/G0 phase, even at 1 μM; however, it caused a concentration-dependent increase in the G2/M cell population (Figure 6B,C). Furthermore, the sub-G1 phase did not appear in LTED or MCF-7 cells exposed to 2-MeO-E2 (Figure 6B), suggesting that 2-MeO-E2 may have an antiproliferative mechanism(s) distinct from that of G-1.

### 3.4. Expression Profiles of β-Tubulin and Its Isotype in MCF-7 and LTED Cells

**Figure 7:**
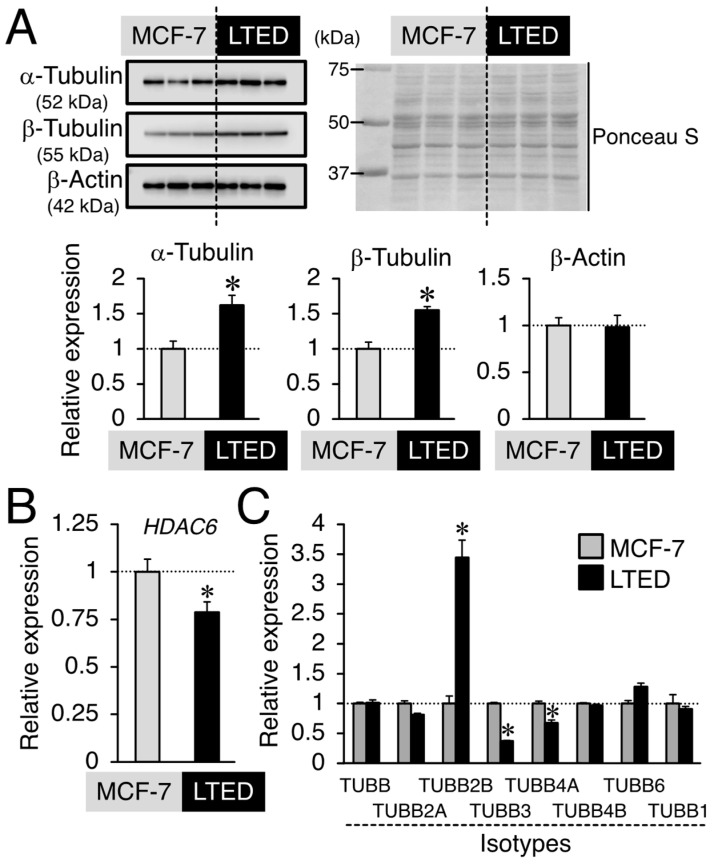
Expression profiles of β-tubulin and its isotype in MCF-7 and LTED cells. (**A**) Basal α-tubulin, β-tubulin, and β-actin protein expression in MCF-7 and LTED cells. (**Upper left panel**) Western blot analysis was performed using antibodies specific for α-tubulin, β-tubulin, and β-actin, respectively. (**Upper right panel**) Ponceau S staining was performed to indicate total protein loading in each lane. (**Lower panel**) The band intensity was quantified using the ImageJ 1.46r software. Data are presented as the mean ± S.E. (*n* = 3) of the fold induction from MCF-7 cells. Significant differences (by Student’s *t*-test) to MCF-7 cells are marked with asterisks (* *p* < 0.05). (**B**,**C**) mRNA expression of Basal *histone deacetylase 6* (*HDAC6*) (**B**) and *β-tubulin* (*TUBB*) isotypes (**C**) in MCF-7 and LTED cells. Data are presented as the mean ± S.E. (*n* = 6) of the fold induction from MCF-7 cells. Significant differences (by Student’s *t*-test) to MCF-7 cells are marked with asterisks (* *p* < 0.05).

We investigated the possible targets underlying the 2-MeO-E2-mediated inhibition of LTED cell proliferation. G-1 and 2-MeO-E2 are microtubule (i.e., polymers of α/β-tubulin)-targeting agents that bind to the colchicine-binding sites of β-tubulin and exert antiproliferative activity in MCF-7 cells [14]. As shown in Figure 7A, Western blot analysis indicated that a high protein expression of α- and β-tubulins (1.62- and 1.55-fold, respectively, *p* < 0.05), but not β-actin, was detected in LTED cells compared to MCF-7 cells. The post-translational modification of α-tubulin has been suggested to be engaged with the aggressive nature of breast cancer cells [40,41]. It has been reported that in the ERα-positive MCF-7 cells, ligand-dependent activation of ERα can upregulate HDAC6, leading to deacetylation of α-tubulin mediated by HDAC6 [41,42]. When focusing on the *HDAC6* mRNA expression between MCF-7 and LTED cells, LTED cells that largely display ligand-independent ERα activation [6,32] showed reduced *HDAC6* levels (~0.79-fold, *p* < 0.05) in comparison to MCF-7 cells (Figure 7B), indicating that the involvement of ERα in the HDAC6/α-tubulin axis might not be high in LTED cells. Additionally, the altered expression of the isotype has been suggested to be involved in resistance to chemotherapy [22,23]. Hereafter, we focused on the β-tubulins and investigated the expression profile of β-tubulin isotypes expressed in MCF-7 and LTED cells. Real-time PCR analysis revealed that compared with MCF-7 cells, a low expression of *TUBB3* and *TUBB4A* (*p* < 0.05) but a significantly higher expression of *TUBB2B* (3.44-fold, *p* < 0.05) was detected in LTED cells (Figure 7C). Thus, among β-tubulin isotypes, the *TUBB2B* gene is a possible target of 2-MeO-E2-mediated antiproliferation in LTED cells.

### 3.5. Effects of siTUBB2B on the Antiproliferation by 2-MeO-E2 in LTED Cells

**Figure 8:**
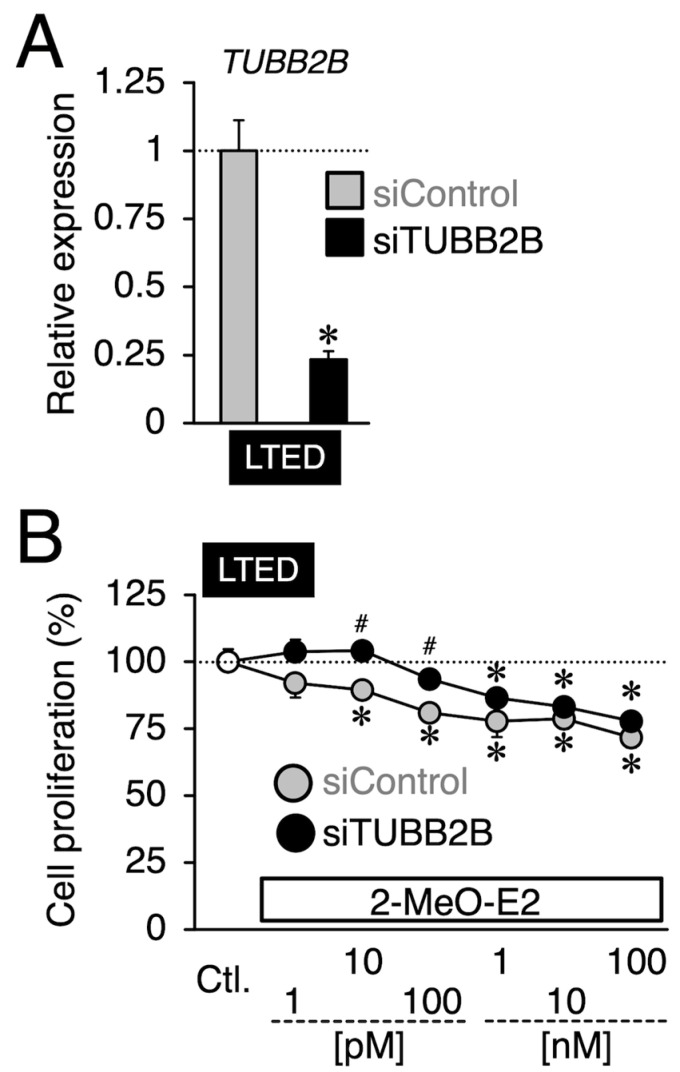
Effects of TUBB2B siRNA (siTUBB2B) on antiproliferation by 2-MeO-E2 against LTED cells. (**A**) mRNA expression of *TUBB2B* in LTED cells transfected with control siRNA (siControl) or siTUBB2B. Data are presented as the mean ± S.E. (*n* = 3) of the fold induction from the siControl-transfected group. Significant differences (by Student’s *t*-test) to the siControl-transfected group are marked with an asterisk (* *p* < 0.05). (**B**) LTED cells were transfected with siControl or siTUBB2B, followed by treatment with 2-MeO-E2 (1 pM to 100 nM). Data are presented as the mean ± S.E. (*n* = 6) percentage of the vehicle-treated control. Significant differences (by two-way ANOVA, followed by Tukey–Kramer’s post hoc test) as compared with the vehicle-treated control and compared with the siControl-transfected group are marked with asterisks (* *p* < 0.05) and hashes (^#^ *p* < 0.05), respectively.

Given that TUBB2B is an antiproliferative target evoked by 2-MeO-E2 in LTED cells, TUBB2B siRNA (siTUBB2B) treatment would somewhat restore the antiproliferative effects of 2-MeO-E2 on LTED cells. We first investigated the effects of siTUBB2B on the basal proliferation of LTED cells. Treatment with siTUBB2B resulted in reduced proliferation (87%, *p* < 0.05) compared to the control siRNA-treated (siControl) group (Supplementary Figure S3), implying the functional role of TUBB2B in the proliferation of LTED cells. To confirm the above-mentioned possibility, we applied 2-MeO-E2 to siTUBB2B-treated LTED cells maximally at 100 nM based on the results in Figure 3B, which revealed selective antiproliferation by 2-MeO-E2 against LTED cells below 100 nM. As shown in Figure 8A, the treatment with siTUBB2B caused a 0.23-fold decrease in *TUBB2B* compared to that in the siControl group. Compared with the siControl group, siTUBB2B restored the reduction in cell viability caused by 2-MeO-E2 over almost the same concentration range (at 10 and 100 pM; *p* < 0.05), although the rescue effect of siTUBB2B was lower, particularly at higher concentrations of 2-MeO-E2 (>10 nM) (Figure 8B).

### 3.6. Effects of G-1 on the 2-MeO-E2-Mediated Antiproliferation in LTED Cells

**Figure 9:**
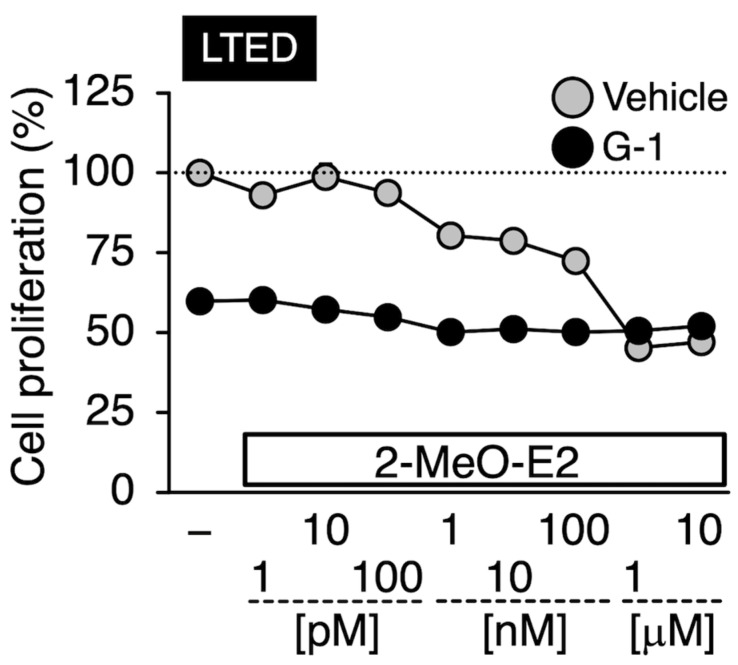
Effects of G-1 on the 2-MeO-E2-mediated antiproliferation in LTED cells. LTED cells were treated with 2-MeO-E2 (1 pM to 10 μM) in the absence or presence of 1 μM G-1 for 48 h. The control sample was treated with vehicle only. Data are presented as the mean ± S.E. (*n* = 6) percentage of the vehicle-treated control.

The binding potential of G-1 to β-tubulin is stronger than that of 2-MeO-E2 [14,16]. Therefore, we investigated whether adding G-1 abrogated the 2-MeO-E2-mediated inhibition of LTED cell proliferation. Compared with the control group, 2-MeO-E2-treated cells showed reduced proliferation in a concentration-dependent manner at concentrations up to 10 µM. At 1 μM, G-1 treatment alone reduced the proliferation of LTED cells to approximately 60% of that of the control. However, an additive antiproliferative interaction between 2-MeO-E2 and G-1 was not observed, and the degree of inhibition was comparable to that of the G-1 alone system (Figure 9). Thus, G-1 and 2-MeO-E2 may share a mechanism of action with β-tubulin in LTED cells.

## 4. Discussion

We used two breast cancer cell lines, MCF-7 (a parental cell line for LTED) and LTED cells (a cell model resistant to endocrine therapy). Compared to MCF-7 cells, LTED cells showed a much higher proliferative potential (Figure 2B) and displayed resistance to microtubule-destabilizing agents (paclitaxel, nocodazole, and colchicine) (Figure 4). Additionally, we reported that LTED cells were tolerant to different kinds of established antiproliferative agents, etoposide, LY2835219, and trichostatin A [24]. We sought to identify chemicals that selectively kill LTED cells and focused on two antiproliferative molecules, G-1 and 2-MeO-E2, which can target both GPER1 and β-tubulin to display their antiproliferative activities. Whereas G-1 preferentially inhibited MCF-7 cell proliferation, 2-MeO-E2 exhibited antiproliferative activity in LTED cells (Figure 3). The protein expression analysis of GPER1 and β-tubulin in MCF-7 and LTED cells revealed upregulated β-tubulin but downregulated GPER1 in LTED cells compared with MCF-7 cells (Figure 2A and Figure 7A). Among the *β-tubulin* gene isotypes, the highest expression of *TUBB2B* was detected in LTED cells compared to MCF-7 cells (Figure 7C). The introduction of siTUBB2B restored 2-MeO-E2-driven antiproliferation (Figure 8B), indicating that 2-MeO-E2 is an effective pharmacological modality; that is, the 2-MeO-E2–TUBB2B interaction is a promising candidate for the abrogation of LTED cell proliferation.

We could not detect the sub-G1 phase in LTED or MCF-7 cells exposed to 2-MeO-E2 (Figure 5 and Figure 6), suggesting that 2-MeO-E2 may utilize mechanism(s) distinct from G-1 for the abrogation of breast cancer cell proliferation. In support of this notion, a recent study reported that 2-MeO-E2 induces centrosome declustering in breast cancer cells, resulting in mitotic arrest via multipolar spindle formation [43].

G-1 and 2-MeO-E2 can bind to the colchicine-binding site of β-tubulin, and G-1 displays a stronger binding potential to the tubulin site than 2-MeO-E2 [14,44]. Compared to the results in which LTED cells were singly treated with 2-MeO-E2, the antiproliferative effects of 2-MeO-E2 were completely blocked by the co-introduction of G-1 (1 μM) (Figure 9). Given that 2-MeO-E2 targets β-tubulin to exert its antiproliferative activity in LTED cells, the binding potency itself might not be essential for the action. Furthermore, after 2-MeO-E2 interaction with β-tubulin (TUBB2B), the downstream antiproliferative signaling pathways might differ between G-1 and 2-MeO-E2. Although little is known about the functional participation of the TUBB2B subtype in breast cancer, including endocrine therapy-resistant ER-positive breast cancer, TUBB2B can function as an oncogene in hepatocellular carcinoma and plays a role in promoting cell proliferation [45]. Among the β-tubulin isotypes, significant upregulation of *TUBB2B* was noted in LTED cells compared to parental MCF-7 cells, suggesting the possibility that TUBB2B can play a common biological role in cancer cells. Although 2-MeO-E2 was suggested to have no antiproliferative effect in vivo and in vitro (on MCF-7 cells) because of its low stability [46,47], the antiproliferative effects of 2-MeO-E2 on LTED cells were not only detected but also prolonged in a time-dependent manner for up to 96 h, with lower IC_50_ values of 0.55 ± 0.02 and 0.40 ± 0.02 μM observed at 72 and 96 h, respectively (Figure 3 and Supplemental Figure S1). Although further research is required, this study strongly suggests that 2-MeO-E2 can be used as a prototype for developing anticancer agents against endocrine therapy-resistant breast cancers.

## 5. Conclusions

LTED cells were used as an experimental model of endocrine therapy-resistant ER-positive breast cancer. Compared to the parental MCF-7 cells, LTED cells display highly aggressive cancerous behavior. Due to the acquisition of resistance, effective modalities for abrogating LTED cell proliferation are required. In this study, we focused on several tubulin-inhibitory drugs, including paclitaxel, nocodazole, colchicine, G-1, and 2-MeO-E2. It was demonstrated that 2-MeO-E2 selectively suppresses the proliferation of LTED cells compared to that of MCF-7 cells (i.e., IC_50_ values: 0.93 μM for LTED cells vs. 6.79 μM for MCF-7 cells). Among the β-tubulin isotypes, LTED cells display the highest upregulated expression of *TUBB2B* compared to parental MCF-7 cells. In the G2/M phase, 2-MeO-E2 arrested LTED cells, in conjunction with a reduced number of cells in the S phase, and targeted TUBB2B for antiproliferative activity. Our findings suggest that TUBB2B-targeting 2-MeO-E2 is a potential candidate molecule for preventing LTED cell proliferation. However, further studies are required to assess whether 2-MeO-E2 abrogates LTED cell-driven breast cancer progression in vivo.

## Data Availability

Not applicable.

## Supplementary Materials

The following supporting information can be downloaded from https://www.mdpi.com/article/10.3390/cimb45090464/s1: Figure S1: Effect of 2-methoxyestradiol (2-MeO-E2) on long-term estrogen-deprived (LTED) cell proliferation; Figure S2: Effects of G-1 on MCF-7 and LTED cell morphology; and Figure S3: Effects of TUBB2B siRNA (siTUBB2B) on the proliferation of LTED cells.

## References

[1] Early Breast Cancer Trialists’ Collaborative Group (EBCTCG) (2015). Aromatase inhibitors versus tamoxifen in early breast cancer: Patient-level meta-analysis of the randomised trials. Lancet.

[2] Katzenellenbogen B.S., Kendra K.L., Norman M.J., Berthois Y. (1987). Proliferation, hormonal responsiveness, and estrogen receptor content of MCF-7 human breast cancer cells grown in the short-term and long-term absence of estrogens. Cancer Res..

[3] Jeng M.H., Shupnik M.A., Bender T.P., Westin E.H., Bandyopadhyay D., Kumar R., Masamura S., Santen R.J. (1998). Estrogen receptor expression and function in long-term estrogen-deprived human breast cancer cells. Endocrinology.

[4] Chan C.M.W., Martin L.-A., Johnston S.R.D., Ali S., Dowsett M. (2002). Molecular changes associated with the acquisition of oestrogen hypersensitivity in MCF-7 breast cancer cells on long-term oestrogen deprivation. J. Steroid Biochem. Mol. Biol..

[5] Martin L.A., Ribas R., Simigdala N., Schuster E., Pancholi S., Tenev T., Gellert P., Buluwela L., Harrod A., Thornhill A. (2017). Discovery of naturally occurring *ESR1* mutations in breast cancer cell lines modelling endocrine resistance. Nat. Commun..

[6] Hirao-Suzuki M., Takeda S., Kodama Y., Takiguchi M., Toda A., Ohara M. (2020). Metalloestrogenic effects of cadmium are absent in long-term estrogen-deprived MCF-7 cells: Evidence for the involvement of constitutively activated estrogen receptor α and very low expression of G protein-coupled estrogen receptor 1. Toxicol. Lett..

[7] Carmeci C., Thompson D.A., Ring H.Z., Francke U., Weigel R.J. (1997). Identification of a gene (GPR30) with homology to the G-protein-coupled receptor superfamily associated with estrogen receptor expression in breast cancer. Genomics.

[8] Bologa C.G., Revankar C.M., Young S.M., Edwards B.S., Arterburn J.B., Kiselyov A.S., Parker M.A., Tkachenko S.E., Savchuck N.P., Sklar L.A. (2006). Virtual and biomolecular screening converge on a selective agonist for GPR30. Nat. Chem. Biol..

[9] Albanito L., Madeo A., Lappano R., Vivacqua A., Rago V., Carpino A., Oprea T.I., Prossnitz E.R., Musti A.M., Ando S. (2007). G protein-coupled receptor 30 (GPR30) mediates gene expression changes and growth response to 17β-estradiol and selective GPR30 ligand G-1 in ovarian cancer cells. Cancer Res..

[10] Lucki N.C., Sewer M.B. (2011). Genistein stimulates MCF-7 breast cancer cell growth by inducing acid ceramidase (*ASAH1*) gene expression. J. Biol. Chem..

[11] Vivacqua A., Romeo E., De Marco P., De Francesco E.M., Abonante S., Maggiolini M. (2012). GPER mediates the egr-1 expression induced by 17β-estradiol and 4-hydroxitamoxifen in breast and endometrial cancer cells. Breast Cancer Res. Treat..

[12] Scaling A.L., Prossnitz E.R., Hathaway H.J. (2014). GPER mediates estrogen-induced signaling and proliferation in human breast epithelial cells and normal and malignant breast. Horm. Cancer.

[13] Santolla M.F., Avino S., Pellegrino M., De Francesco E.M., De Marco P., Lappano R., Vivacqua A., Cirillo F., Rigiracciolo D.C., Scarpelli A. (2015). SIRT1 is involved in oncogenic signaling mediated by GPER in breast cancer. Cell Death Dis..

[14] Lv X., He C., Huang C., Hua G., Wang Z., Remmenga S.W., Rodabough K.J., Karpf A.R., Dong J., Davis J.S. (2017). G-1 inhibits breast cancer cell growth via targeting colchicine-binding site of tubulin to interfere with microtubule assembly. Mol. Cancer Ther..

[15] Wang C., Lv X., He C., Hua G., Tsai M.-Y., Davis J.S. (2013). The G-protein-coupled estrogen receptor agonist G-1 suppresses proliferation of ovarian cancer cells by blocking tubulin polymerization. Cell Death Dis..

[16] D’Amato R.J., Lin C.M., Flynn E., Folkman J., Hamel E. (1994). 2-Methoxyestradiol, an endogenous mammalian metabolite, inhibits tubulin polymerization by interacting at the colchicine site. Proc. Natl. Acad. Sci. USA.

[17] Prossnitz E.R., Arterburn J.B. (2015). International union of basic and clinical pharmacology. XCVII. G protein-coupled estrogen receptor and its pharmacologic modulators. Pharmacol. Rev..

[18] Cavalieri E., Chakravarti D., Guttenplan J., Hart E., Ingle J., Jankowiak R., Muti P., Rogan E., Russo J., Santen R. (2006). Catechol estrogen quinones as initiators of breast and other human cancers: Implications for biomarkers of susceptibility and cancer prevention. Biochim. Biophys. Acta.

[19] Mueck A.O., Seeger H., Huober J. (2004). Chemotherapy of breast cancer-additive anticancerogenic effects by 2-methoxyestradiol?. Life Sci..

[20] Zhu B.T., Conney A.H. (1998). Is 2-methoxyestradiol an endogenous estrogen metabolite that inhibits mammary carcinogenesis?. Cancer Res..

[21] Ludueńa R.F., Shooter E.M., Wilson L. (1977). Structure of the tubulin dimer. J. Biol. Chem..

[22] Ranganathan S., Benetatos C.A., Colarusso P.J., Dexter D.W., Hudes G.R. (1998). Altered β-tubulin isotype expression in paclitaxel-resistant human prostate carcinoma cells. Br. J. Cancer.

[23] Kavallaris M., Burkhart C.A., Horwitz S.B. (1999). Antisense oligonucleotides to class III β-tubulin sensitize drug-resistant cells to taxol. Br. J. Cancer.

[24] Takeda S., Hirao-Suzuki M., Yamagishi Y., Sugihara T., Kaneko M., Sakai G., Nakamura T., Hieda Y., Takiguchi M., Okada M. (2021). Effects of the ethanol extract of *Neopyropia Yezoensis*, cultivated in the Seto Inland Sea (Setonaikai), on the viability of 10 human cancer cells including endocrine therapy-resistant breast cancer cells. Fundam. Toxicol. Sci..

[25] Sakai G., Hirao-Suzuki M., Koga T., Kobayashi T., Kamishikiryo J., Tanaka M., Fujii K., Takiguchi M., Sugihara N., Toda A. (2022). Perfluorooctanoic acid (PFOA) as a stimulator of estrogen receptor-negative breast cancer MDA-MB-231 cell aggressiveness: Evidence for involvement of fatty acid 2-hydroxylase (FA2H) in the stimulated cell migration. J. Toxicol. Sci..

[26] Takeda S., Hirao-Suzuki M., Shindo M., Aramaki H. (2022). (–)-Xanthatin as a killer of human breast cancer MCF-7 mammosphere cells: A comparative study with salinomycin. Curr. Issues Mol. Biol..

[27] Duong V., Bret C., Altucci L., Mai A., Duraffourd C., Loubersac J., Harmand P.O., Bonnet S., Valente S., Maudelonde T. (2008). Specific activity of class II histone deacetylases in human breast cancer cells. Mol. Cancer Res..

[28] Narvi E., Jaakkola K., Winsel S., Oetken-Lindholm C., Halonen P., Kallio L., Kallio M.J. (2013). Altered TUBB3 expression contributes to the epothilone response of mitotic cells. Br. J. Cancer.

[29] Saussede-Aim J., Matera E.-L., Ferlini C., Dumontet C. (2009). β3-Tubulin is induced by estradiol in human breast carcinoma cells through an estrogen-receptor dependent pathway. Cell Motil. Cytoskelet..

[30] Azumi M., Yoshie M., Nakachi N., Tsuru A., Kusama K., Tamura K. (2022). Stathmin expression alters the antiproliferative effect of eribulin in leiomyosarcoma cells. J. Pharmacol. Sci..

[31] Li W., Zhai B., Zhi H., Li Y., Jia L., Ding C., Zhang B., You W. (2014). Association of ABCB1, β tubulin I, and III with multidrug resistance of MCF7/DOC subline from breast cancer cell line MCF7. Tumour Biol..

[32] Hirao-Suzuki M., Takiguchi M., Yoshihara S., Takeda S. (2023). Repeated exposure to 4-methyl-2,4-bis(4-hydroxyphenyl)pent-1-ene (MBP) accelerates ligand-independent activation of estrogen receptors in long-term estradiol-deprived MCF-7 cells. Toxicol. Lett..

[33] Wei W., Chen Z.-J., Zhang K.-S., Yang X.-L., Wu Y.-M., Chen X.-H., Huang H.-B., Liu H.-L., Cai S.-H., Du J. (2014). The activation of G protein-coupled receptor 30 (GPR30) inhibits proliferation of estrogen receptor-negative breast cancer cells in vitro and in vivo. Cell Death Dis..

[34] Girgert R., Emons G., Gründker C. (2012). Inactivation of GPR30 reduces growth of triple-negative breast cancer cells: Possible application in targeted therapy. Breast Cancer Res. Treat..

[35] Weißenborn C., Ignatov T., Ochel H.-J., Costa S.D., Zenclussen A.C., Ignatova Z., Ignatov A. (2014). GPER functions as a tumor suppressor in triple-negative breast cancer cells. J. Cancer Res. Clin. Oncol..

[36] Weißenborn C., Ignatov T., Poehlmann A., Wege A.K., Costa S.D., Zenclussen A.C., Ignatov A. (2014). GPER functions as a tumor suppressor in MCF-7 and SK-BR-3 breast cancer cells. J. Cancer Res. Clin. Oncol..

[37] Lu Y., Chen J., Xiao M., Li W., Miller D.D. (2012). An overview of tubulin inhibitors that interact with the colchicine binding site. Pharm. Res..

[38] Mills J.C., Stone N.L., Erhardt J., Pittman R.N. (1998). Apoptotic membrane blebbing is regulated by myosin light vhain phosphorylation. J. Cell Biol..

[39] van den Brand A.D., Villevoye J., Nijmeijer S.M., van den Berg M., van Duursen M.B.M. (2019). Anti-tumor properties of methoxylated analogues of resveratrol in malignant MCF-7 but not in non-tumorigenic MCF-10A mammary epithelial cell lines. Toxicology.

[40] Hubbert C., Guardiola A., Shao R., Kawaguchi Y., Ito A., Nixon A., Yoshida M., Wang X.-F., Yao T.-P. (2002). HDAC6 is a microtubule-associated deacetylase. Nature.

[41] Azuma K., Urano T., Horie-Inoue K., Hayashi S., Sakai R., Ouchi Y., Inoue S. (2009). Association of estrogen receptor α and histone deacetylase 6 causes rapid deacetylation of tubulin in breast cancer cells. Cancer Res..

[42] Yoshida N., Omoto Y., Inoue A., Eguchi H., Kobayashi Y., Kurosumi M., Saji S., Suemasu K., Okazaki T., Nakachi K. (2004). Prediction of prognosis of estrogen receptor-positive breast cancer with combination of selected estrogen-regulated genes. Cancer Sci..

[43] El-Zein R., Thaiparambil J., Abdel-Rahman S.Z. (2020). 2-Methoxyestradiol sensitizes breast cancer cells to taxanes by targeting centrosomes. Oncotarget.

[44] Dumontet C., Jordan M.A. (2010). Microtubule-binding agents: A dynamic field of cancer therapeutics. Nat. Rev. Drug Discov..

[45] Wang X., Shi J., Huang M., Chen J., Dan J., Tang Y., Guo Z., He X., Zhao Q. (2023). TUBB2B facilitates progression of hepatocellular carcinoma by regulating cholesterol metabolism through targeting HNF4A/CYP27A1. Cell Death Dis..

[46] Mooberry S.L. (2003). New insights into 2-methoxyestradiol, a promising antiangiogenic and antitumor agent. Curr. Opin. Oncol..

[47] Sutherland T.E., Schuliga M., Harris T., Eckhardt B.L., Anderson R.L., Quan L., Stewart A.G. (2005). 2-Methoxyestradiol is an estrogen receptor agonist that supports tumor growth in murine xenograft models of breast cancer. Clin. Cancer Res..

