# Supplementary material for: 2-Methoxyestradiol as an Antiproliferative Agent for Long-Term Estrogen-Deprived Breast Cancer Cells
Source: Curr Issues Mol Biol. 2023 Sep 9;45(9):7336–51. doi: 10.3390/cimb45090464 (PMC10527823; doi:10.3390/cimb45090464)
Supplement: Supplementary file 1 [file cimb-45-00464-s001.zip › cimb-2526369-supplementary.pdf]

Supplementary figures

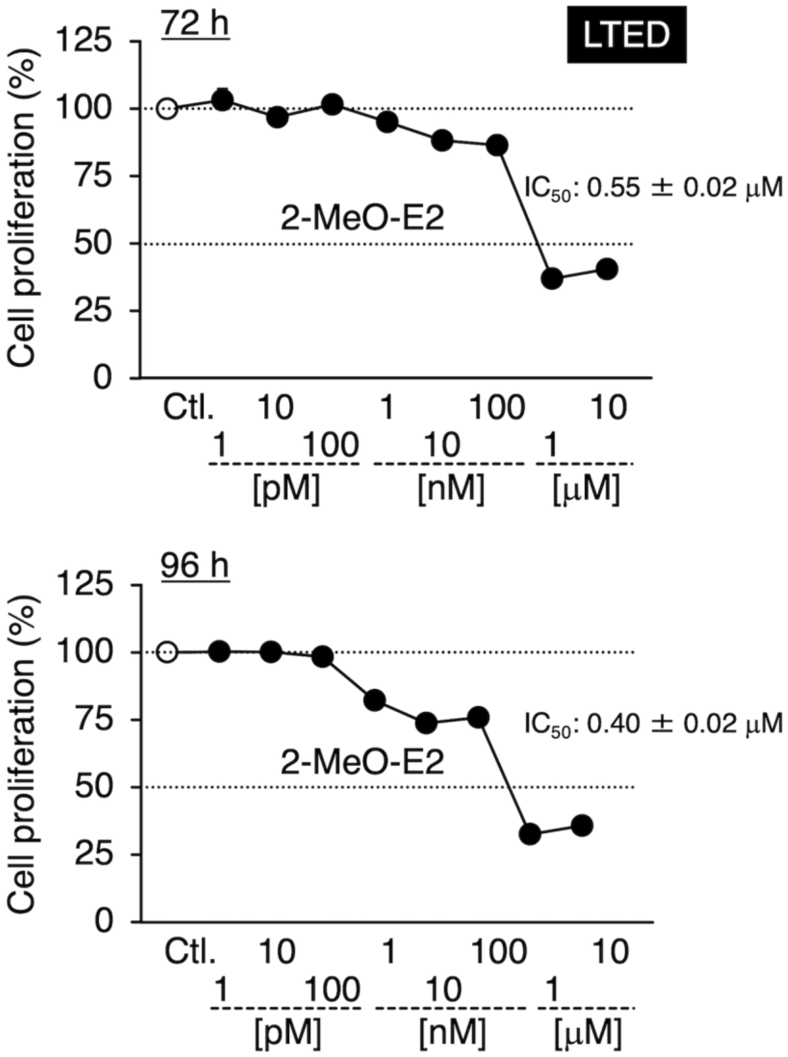

Figure S1. Effect of 2-methoxyestradiol (2-MeO-E2) on long-term estrogen-deprived (LTED) cell proliferation. LTED cells were treated with 2-MeO-E2 (1 pM to 10 μM) for 72 (**upper panel**) or 96 h (**lower panel**). Control samples (controls) cells were treated with the vehicle. Data are presented as the mean ± S.E. (n = 6) percentage of the vehicle-treated control.

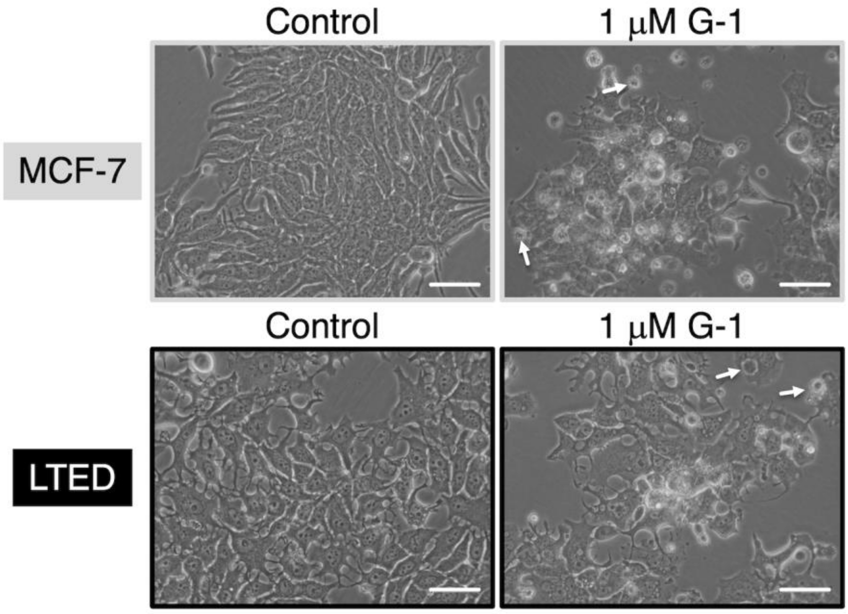

Figure S2. Effects of G-1 on MCF-7 and LTED cell morphology. Morphology of MCF-7 and LTED cells treated with 1 μM G-1 for 48 h. The control sample was treated with the vehicle. Representative images are shown. Images were acquired at 400× magnification. The scale bar represents 50 μm. Some cell blebs are indicated by white arrows.

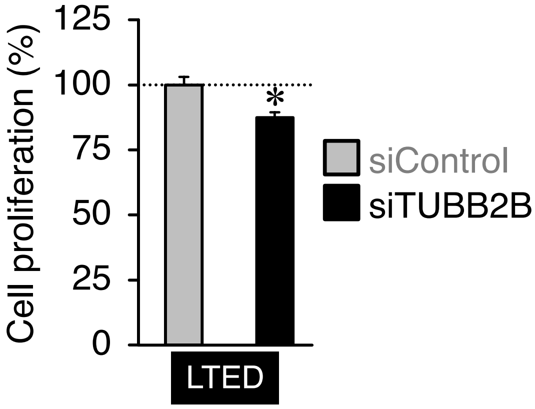

Figure S3. Effects of TUBB2B siRNA (siTUBB2B) on the proliferation of LTED cells. LTED cells were transfected with control siRNA (siControl) or siTUBB2B. Data are presented as the mean ± S.E. (n = 6) percentage of the siControl-transfected group. Significant differences (by Student's t-test) to the siControl-transfected group are marked with an asterisk (*$P < 0.05$).
